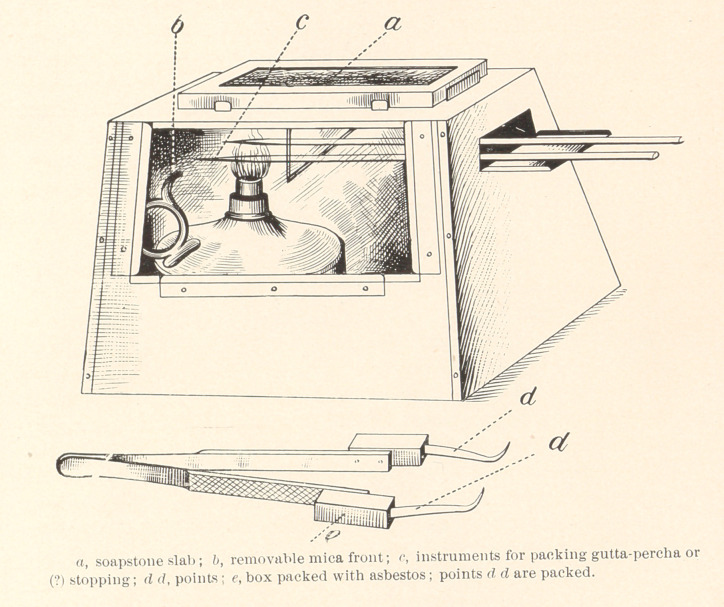# New York Odontological Society

**Published:** 1893-04

**Authors:** 

**Affiliations:** New York Odontological Society


### NEW YORK ODONTOLOGICAL SOCIETY.

A REGULAR meeting of the New York Odontological Society was held on Tuesday evening, January 17, 1893, at the New York Academy of Medicine, No. 17 West Forty-third Street, New York City, with the Vice-President, Dr. Brockway, in the chair.

The minutes of the previous meeting were read and approved.

INCIDENTS OF PRACTICE AND CASUAL COMMUNICATIONS.

*Dr. George Allan.*—For some time I have thought that this part of our proceedings ought to receive a great deal more attention than it has in the past. So I decided this evening to bring before the Society two or three little things that I have found useful in my practice, and let you judge them for what they are worth, and decide whether they are good or not. The first thing is a celluloid sheet; I have used it instead of a towel or oil-cloth for the top of my table and my bracket. It is absolutely clean, is wiped off without the slightest difficulty, and it seems to meet all the requirements for a covering of that kind. The greatest objection to it is that if you happen to let a match fall upon it, it will burn very rapidly. It will warp a little if the sun shines upon it, but practically it has no serious objection, and I would not be without it. It is cheap and economical, and a sheet will last two or three years. If you let sulphate of iron or iodine fall upon it, it will, of course, stain; but for neatness and cleanliness I know of nothing that is its equal. I have a sheet of it here. I think enough to cover a bracket would cost about sixty or seventy cents. It comes by the yard or by the sheet, in all sizes, shapes, and colors. You can get it at the Celluloid Company in Newark; they have an office in New York, somewhere on Broadway.

The next little appliance is a pair of pincers for soldering. You take the ordinary pincers and hold a piece of gold over a Bunsen burner, and, unless you are very quick, your fingers will get very hot and you must drop it. I had a pair of pincers made with each of the points set in a little box of asbestos. I find it very valuable and satisfactory. You can leave it in the burner as long as you please, and the heat will not affect it; it is a good thing both in the office and in the laboratory.

Some years ago, when I was in Boston in Dr. Shepard's office, I found he heated his gutta-percha by placing one end of a piece of soapstone in or close to a Bunsen burner. In use he placed the gutta-percha on the cooler end of the stone, and then passed it down to the heated end, and in this way controlled the heat perfectly. The arrangement was very effective, but had some objections. I got up a little oven which meets every want of the dentist for warming gutta-percha. When in use, you light the spirit-lamp and place it under the end of the stone; at the same time the instruments to be used are passed through the opening at the end of the oven, over the wire loop inside to the flame of the lamp, so that

*a*, soapstone slab; *b*, removable mica front; *c*, instruments for packing gutta-percha or (?) stopping; *d d*, points; *e*, box packed with asbestos; points *d d* are packed.

the instruments to be used and the gutta-percha will be warming at the same time. The slab, you will notice, is hollowed out, so that the small pieces of gutta-percha will not roll off. The stone heats gradually, and the amount of heat required can be regulated to a nicety by the size of the flame on the portion of the stone the gutta-percha is placed on. This little mica is put there so you can see the flame and tell where you place your points, and get them as near the flame as you desire. The purpose of the mica is to prevent the flame from blowing out, if you are working in summer with the doors open and are in a draught; of course, in winter it is not necessary. You can take out the instrument and put it directly in the flame. There is no danger of overheating the gutta-percha, as I said before; and while you are getting your cavity ready, you are at the same time getting your instruments heated to the right temperature. There is a little arrangement advertised by the Whites, I think, which is called Dr. Howe's idea. He has a slab of soapstone with a handle attached. You pass it into the flame and heat it in that way; but this has all the advantages of that, and a great many in addition. It certainly is very effective and very convenient.

*Dr. Littig.*—Is the dry heat better than the moist?

*Dr. Allan.*—I have found it so. You get any heat you desire. I use Dr. Flagg's gutta-percha, and he has two or three different varieties. You require a greater range of heating surface to warm it. It seems to meet all the requirements for that purpose.

About two weeks ago I had an accident, and it set me thinking, and I determined I would not have the same happen again in my practice. About two years ago I exposed a pulp, capped it, protected it, and, as the crown was decayed, I put a Richmond crown on. The tooth broke about a month ago, and, in my haste in getting the root ready, I chipped out the little piece of oxyphosphate that I had placed over my capping, and re-exposed the pulp. If I had referred to my book I would have noticed at once that the pulp had been capped, and yet there was not the slightest indication about the tooth that it had been done; it seemed to me that some method ought to be adopted by which we could tell the condition of things. There is no better way than by having the material itself tell the story. In other words, I would have my gutta-percha or oxyphosphate, whatever I used, a bright red color. If that were generally practised, no dentist would ever commit the piece of folly that I did, of needlessly re-exposing a pulp that had once been exposed by cutting away the cover. By mixing a little vermilion with the gutta-percha or oxyphosphate you get a perfectly red filling-material. This, then, I proposed to use immediately over the cap covering the exposed pulp. A tooth so treated tells its own story, and the danger of re-exposing a pulp once exposed, treated, and protected, will be reduced to a minimum. In excavating, if this plan should be generally adopted, the moment one sees that red signal he would know there was an exposed pulp, whether he had done the work himself or some one else had. I added some of the vermilion with the oxyphosphate powder, and found that it did not interfere with the setting when mixed with the acid. I do not see why that should not be a useful point in practice. We would then have a double record,—the record-book and the material itself. If it was always used for that purpose, we could not make the mistake I did. When I first thought of coloring the filling material, I wrote to Dr. Kirk and asked him what would be a negative powder that I could incorporate into the oxyphosphate or gutta-percha, and he wrote back suggesting vermilion. Fortunately, I had the vermilion in the office, and immediately tried it with this result.

Here is another little appliance. Most of you have used disinfecting and germicide fluids, and have them on your desk for use. You want something to hold them. This is handy, cleanly, and at the same time will keep the fluid from evaporating or the dust from getting in. These jars were made for preserving pathological specimens. The cap has a circular groove ground into it to place over in that way. It is practically a glass-stopped bottle, but there is no neck to the bottle. The fluid does not evaporate, the dust does not get into it, and it is always handy for use. As I stated in a short paper I read before the Boston Academy, I use bichloride in the strength of one to two thousand. I dip my instruments in it, fill my syringe with it, and it keeps the mouth-mirror and the syringe clean, as well as most of my instruments. I dip my scaling instruments in it, and I find it is very effective, and, having it in this cleanly shape before me, it saves time. These jars come in three or four sizes, and you can have them in almost any shape you please. From the shape of these (being an ordinary tumbler-shape) they are easily kept clean. You get these at George Wollman's, 116 Fulton Street.

A year or two ago I commenced practising with diamond powder for polishing enamel, and for a variety of purposes. There is about two dollars and fifty cents' worth in this little bottle that I have here; a little diamond powder goes a great way. I use a very little of it mixed with Hindostan powder, and find it very effective. I use it with a copper point, or take an old burr, heat it and soften it, and take off the burr part so I have simply a rounded part, dip it in oil of cloves or oil of wintergreen and use it, and it is astonishing how rapidly it works, and what a bright, clean surface it gives.

*Dr. Delos Palmer.*—Is that diamond powder that you have in that bottle?

*Dr. Allan.*—Yes; it is cheaper than you would think, because you mix it with the other powder, and that saves it to a great extent, and it saves time enough to make the value of the diamond-dust a very small matter. In reference to the Hindostan powder, when the engine was first introduced it was a question what kind of stones would be most useful for polishing. When I was at college I spent a great deal of time grinding some lenses, and I found that R. B. Tolles, who was then one of the best manufacturers of microscope objectives we had, used Hindostan stone for grinding and polishing his lenses. I met him afterwards, and, in speaking of the matter, he said it was one of the most valuable powders he had ever used. The ordinary Hindostan powder will scratch, but the S. S. White Company, at my suggestion, have made two or three grades by precipitation, and the finer ones will cut and polish without scratching. Believe me, it is a matter of no small moment to finish off a filling or roughened tooth-surface without scratching. I believe these different grades of Hindostan powder are the best powders in the market for dental use.

Dr. R. I. Blakeman then read a short paper entitled, "Filling Roots with Gutta-Percha dissolved in Chloroform."

(For Dr. Blakeman's paper, see page 267.)

DISCUSSION.

*Dr. Perry.*—While approving very heartily this method of treatment, I would like to suggest, instead of the point of gutta-percha (which I have used for many years myself, and which I presented to the profession), for plunging into this softened mass, a point of gold wire, sharpened down to fit as nearly as possible the canal of the root, the larger blunt end of which is notched to make it possible to get hold of it for easy removal. I think one point that this gentleman has made is very good, and that is the ease with which the gutta-percha points can be removed. It is not very nice to cut and pull at a tender root, and I have always maintained that it is a good thing in a filling-material that it can be removed easily.

Those gold points are easily removed, and then very easy access is made to the end of the root, particularly by chloroform, as the essayist said. So you can be as gentle as a kitten if the time ever comes to remove them. Of course, we all hope we never will have to remove them. The penetrating and searching effect of that material, without doubt, makes one of the best fillings we ever have had. The only weak point is that it has not, as Dr. Howe several years ago pointed out, the antiseptic property, and when removed there will be a bad odor about it which shows that there is germ life present. The oxychloride of zinc can never be as nicely and as completely adjusted to all the inner parts of the tooth as that material, although we know it does not decay. If we want to be extremely fine, our work should not consist in filling the root-canal alone; we should fill as far as we can the tubuli which branch off from it. Of course, that is carrying the operation rather far, but still we can do it sometimes. Touch the dentine of an old tooth with a rapidly revolving burr, and you will stir up a bad odor. If that condition of a tooth can be prevented, it is very desirable.

*Dr. S. E. Davenport.*—I hoped that Dr. Blakeman would give the history of a case which I had the privilege of seeing in his chair, but as he has not, I would like to say a few words about it. A lower bicuspid in a gentleman's mouth had abscessed a number of years ago, and when Dr. Blakeman first saw it the apex of the root had become denuded and stuck out through an opening in the gum. Dr. Blakeman thought best to trim off the end of the root, and in doing so the side of the pulp-canal was opened into for a short distance. This canal was smaller than is usual in bicuspids of that size, and when the root was filled in the manner described it was an object-lesson, for we do not often have the privilege of seeing both ends of the tooth during such an operation. The solution almost immediately appeared at the thread-like opening, and in experimenting it was found that the least pressure upon the gutta-percha in the pulp-chamber would cause the solution to project from the opening near the apex.

*Dr. Northrop.*—From the essayist's paper this evening you would judge that filling the root-canals is a very nice, pleasant operation. I tried it quite a good deal twenty-five years ago, and I would like to ask if the essayist ever encounters any difficulty in forcing this gutta-percha up to the apex of the root without clinging to his instruments and making trouble. From the paper you would suppose that all we need to do is to wipe out the root, put a little soft gutta-percha in it, and a plunger carries it right up. Does he never have the disagreeable result of the gutta-percha pulling out and making a mess of the whole thing? Does it always go up easily and neatly? It is a delightful thing when you get it there, but is it always as easy to do as he describes? I would like to know very much.

*Dr. Blakeman.*—I did not mean to convey the idea that this was an enticing operation, but rather to describe a way by which these minute canals could be filled. I have seldom experienced much difficulty in getting this solution into the roots of either the upper or lower teeth. Dr. Perry's suggestion of putting some pure chloroform into the roots before filling them is a good one; in fact, I think it is sometimes necessary with this method.

*Dr. Allan.*—Why do you fill those fine roots?

*Dr. Blakeman.*—I think it is necessary.

*Dr. Allan.*—Why is it necessary?

*Dr. Blakeman.*—I have had a few cases which I thought gave trouble because these fine roots were not filled. My reason for thinking so is that the trouble ceased after the roots were filled.

*Dr. Northrop.*—Will Dr. Allan not continue his remarks, and tell us how he disinfects the fine roots?

*Dr. Allan.*—I intended doing so. The cavity and roots of the tooth under treatment are dried out thoroughly, the rubber dam being first placed over the tooth by the use of bibulous paper and the hot-air syringe. Then the root or roots are cleansed out as thoroughly as possible, using cotton wrapped on broaches, etc. The drying process by these means can be made to extend well up into the roots. A fine platinum wire heated and passed well into the roots is very effective at times, but for the completion of the drying process I depend on alcohol (absolute is the best), which, by its strong affinity for water, will run into the roots, work its way up, and displace the water. By means of broaches passed into the roots, or a wad of cotton pressed over their open ends, this process can be expedited.

*Dr. Smith.*—Does capillary attraction act if one end of the tooth is closed?

*Dr. Allan.*—No; not at all, or only feebly along the sides of the tube, not filling it. Now, the alcohol is not only a powerful germicide itself, but is also a solvent for most of the essential oils, so that the oil will follow the alcohol and be made to take the place of the water or watery mixture originally filling the roots. Chemical affinity also acts powerfully, first in putting the alcohol into the roots, and finally the oil; and we have reason to believe that this last force even takes the alcohol and oil into the dentinal tubes. In the case of celluloid dissolved in alcohol and ether, used to fill the roots, the same procedure is followed, except that in between the alcohol and the solution of collodion a mixture of two parts of ether and one of alcohol is employed. The collodion mixture is miscible in all proportions with this last, but not at all with alcohol.

*Dr. Smith.*—How do you know?

*Dr. Allan.*—I cannot speak positively. I do know, however, that by passing those broaches up and then taking the cotton and using it with the alcohol and essential oils, effective work can be done.

*Dr. Perry.*—Does the tooth give trouble within five years afterwards?

*Dr. Allan.*—I have not used it long enough to say. I have only used the celluloid a little over two years. It is soluble in ether and alcohol, just the same as gutta-percha is in chloroform. So far it has given much satisfaction.

*Dr. Northrop.*—Then I understand that the last speaker depends more on the cooking out than the washing out.

*Dr. Allan.*—I depend on both. They are both very valuable processes.

*The President.*—If there is nothing further to be said on the subject, we will hear from Dr. J. Morgan Howe on the subject of "Erosion."

(For Dr. Howe's paper, see page 241.)

At the conclusion of Dr. Howe's paper, Dr. S. G. Perry followed with one on the same subject.

(For Dr. Perry's paper, see page 249.)

DISCUSSION.

*Dr Perry.*—The paper which I have just read was most hastily prepared, to comply with the request of the chairman of the Executive Committee to lead the discussion on the subject of erosion. It is not in satisfactory form to present to the profession, but I must let it go as it is, and for what it is worth.

Dr. Bulkley is the specialist to whom I applied for treatment of the eczematous trouble, and it was his great book on that disease that I read most carefully and most profitably. He preached the doctrine of open air, exercise, rest, and proper food, and to good purpose, as I have shown.

I called at his office this evening and asked him to come to our meeting to hear my paper. To my gratification he said he would, though it would deprive him of meeting with one of his own societies which is now being held in one of the adjoining rooms. He is present, and I hope will discuss the subject from the physician's stand-point.

*The President.*—We should all be pleased to hear from Dr. Bulkley.

*Dr. Bulkley.*—When Dr. Perry asked me just now to go with him and speak on his paper, I told him that I disliked to do so because I knew nothing of the actual disease of which he wrote, —namely, the erosion of the teeth. However, of the truth of this theory upon which Dr. Perry is talking,—that is, that the uric-acid diathesis has much to do with many changes of the body, even of the teeth, hair, and nails, I am even more convinced than he is.

It is about seven years since Dr. Perry first came to see me professionally, and his case has been a most interesting one, and I can confirm all he has said in regard to the causes of his troubles and their relief by dietary and hygienic measures. He has repeatedly taken a mixture of acetate of potassa, nux vomica, and quassia, but this only aids in the cure, the other elements referred to being the most important.

The liver, as has been said, is the means in the system of utilizing the nitrogen taken in the food. It has several functions, the chief of which is the conversion of the part of the albumen of the food into urea, which is then excreted by the kidneys. If that metamorphosis fails to go far enough it will produce uric acid. Our idea of gout is that the uric acid thus introduced into the blood attacks the phosphate of soda and potash which renders the blood alkaline, and unites with the former in producing urate of soda, which is deposited in the joints. The alkalinity of the blood is due to the sodium and potassium phosphates with the carbonates. When uric acid is produced and poured into the blood, it attacks these phosphates and carbonates and seizes upon the soda and potash there.

In regard now to the part played in the production of erosions of the teeth by this acid state, we know that the uric acid circulates in the blood, and can be obtained in crystals and from the serum of a blister raised on the skin. In certain cases, also, urate of soda is found on the surface of the skin, having been excreted by the sweat-glands.

It seems, therefore, to me quite probable that a secretion from the mucous follicles may contain some of this same uric acid, which may then attack the phosphates of the teeth and so erode them.

This theory of Dr. Perry's seems to me so natural that I can hardly fail to express my perfect coincidence with it.

Now, while we talk of gout in connection with the subject under consideration, we must not confine our thoughts wholly to what is commonly regarded as gout,—namely, inflammation of one or more of the joints of the body, notably the great toe. We know that the gouty state begins long before there are the acute joint-inflammations, and continues in the intervals between the attacks of the same. I think the matter should be spoken of as the "gouty state or habit."

There are many links of the chain reaching far backward; indeed, so far that the first systemic changes are often lost sight of. The process begins with the faulty metabolism, largely in the liver, and is principally due to errors in diet, although there are other elements of causation, such as lead-poisoning, nervous exhaustion, etc., which need not be discussed here.

The one thought I would throw out is that in searching for the acid cause of this erosion of the teeth, we should not content ourselves with merely inquiring if the patient has what he understands to be gout, but look for other evidences of the lithæmic or acid state of the system.

A long train of symptoms indicate gout, and it would not do for any one who is looking at a patient's eroded teeth simply to ask whether they have had gout alone. There are many other indications, some of which are quite as conclusive of the uric-acid diathesis as is gouty joint disease, but I cannot dwell on this here. Such are acid secretions with urinary deposits, neuralgias, palpitations, sick-headaches, and various other symptoms, which must be looked for, and be taken as elements bearing on the case. The simple denial, therefore, of patients having had gout is not sufficient, but other elements must be looked for and rectified if we would rightly remove the difficulty.

In regard to the diet, the point which Dr. Perry has so well brought out is certainly to my mind a very valuable one, and one most worthy of your earnest consideration.

The medical profession is, as you know, running into specialties, and forgetting far too much the broad principles on which all good practice must rest. I find many gentlemen going into the specialties of the eye, the ear, the throat, the skin, gynæcology, etc., and having no regard for the internal conditions of the system, but centring their thoughts and energies far too exclusively upon the organs which they especially treat. I look upon the dental profession as one great specialty in medicine, and I think that those practising it should be physicians in every sense of the word, with broad views, only having a more perfect knowledge of the contents of the mouth, its diseases, and how to remedy them, than of other parts of the body. The fault I find is, as I say, that the *specialists* are too often *exclusivists*, knowing or thinking far too little beyond their own limited field of practice. I think, therefore, that this discussion, in bringing up the matter of diet and hygiene in connection with diseases of the teeth, which have too often before been considered as local affections, is very important indeed. As we go further and further along in our studies as to the relation of one disease to another, we learn a great deal. In regard to skin-diseases, I cannot, at all coincide with my instructors in Vienna, who regard them almost wholly as local affections, and quite ignore constitutional relations and treatment.

As Dr. Perry said in regard to his own case, he was troubled with an eczema which was most positively of acid or gouty origin. I see many cases of this eruption which are partly gouty; others are neurotic, others scrofulous. You ask me why erosions of the teeth occur in some gouty persons, while others much more afflicted may have perfect teeth, and I cannot answer, any more than I could explain why one person has gout with an eczema, and another has gout and has no eczema; or why one gouty person will have bronchitis, while another is free: we only know that such is the case, and that, for reasons which are at present unaccountable, many local lesions in various parts of the body are often caused by systemic states.

It is quite a significant fact that in an adjoining room, where I should have gone to-night if Dr. Perry had not invited me here, there is a discussion with regard to bacteria and their real influence in the production of disease.

There are some who will there maintain that these micro-organisms are really the product, and not the causes, of disease, or at least that they manifest themselves only when proper conditions of system are present. In other words, the medical profession is looking away from the local causes which have been attributed to some diseases, and is again recognizing that the great changes which go on in the body are often of more real import than are local and external factors. Here in dentistry we are looking for diseases of the teeth beyond local causes, to some systemic cause. It is indeed interesting that the two branches of medicine should thus come so closely together.

*Dr. Northrop.*—As I was about to remark, if we accept the theory of Dr. Perry, which I would be happy to do, we could at once rid ourselves of all responsibility, and say to our patients, "Go to your physician," and we could absolve ourselves of troubles that come to us every day, such as sensitive dentine and erosion. I wish his theory might be correct. Two of the worst cases of erosion I ever saw were of a gouty, rheumatic diathesis; but I could give you almost a hundred instances, right in our midst, that are confirmed gouty people who spend almost one-third of their time in their beds and cannot get around, on account of gout, and yet their teeth are perfect. I will be bound that if Dr. Perry will go over his practice, where he will find one case of erosion with gout connected with it he will find one hundred cases of gout without the slightest particle of erosion. I cannot say that it is not uric acid that affects a tooth, because I do not know.

Last night a gentleman came to me with the canine tooth just as if it had been planed off. On the right side, above, the canine tooth was planed straight to an edge, and also the two bicuspids. He has a gouty diathesis. Another had almost all of his teeth in that condition, but before he lost the teeth he died. He did not die of gout, but of Bright's disease. Of course, that was acid in the blood, too. While I should be most happy to accept that as a theory, I cannot and be satisfied that it is a real cause of erosion. I wish I could. Sometimes you will find a little patch on the enamel; sometimes it is the teeth planed off, just as I said before. I would like to say to my patients, "Go to your physician; it is uric acid in your system." I do not see one thing that we can use to cure it, except to go to the physician and have him rid the system of it.

*Dr. Perry.*—If it is not uric acid, what is it?

*Dr. Northrop.*—I don't know; but the cases are so different.

*Dr. Bulkley.*—You see one hundred cases of gout, and only one of them has eczema, and yet we believe that eczema comes from gout.

*Dr. Northrop.*—What evidence have you that eczema comes from gout?

*Dr. Bulkley.*—It yields to gouty treatment alone, and nothing else.

*Dr. Northrop.*—Can you cure gout?

*Dr. Bulkley.*—Certainly; you eliminate the element by the person living correctly, and the gout ceases. You do not eliminate the *tendency* to gout, because the liver is at fault, and, if the person taxes it, the gout will recur.

*Dr. Lord.*—Mr. President, I have brought to the meeting models to show some of the results of the disease under discussion, and will just say a few words about the case, without making much comment as to the cause.

This may be said, I think, to be a case in which the disease has made great havoc with the teeth, and the work appears to be still going on.

The pencil-marks on the models show that eighteen of the remaining twenty-seven teeth are affected,—the most of them very badly, as is shown by the extent of the pencilling. As is seen, the whole of the labial surfaces of eight of the teeth are quite gone; and the models show that the incisors do not come in contact by one-tenth of an inch, and the canines and bicuspids on the right side do not meet by quite a little space, and the corresponding teeth on the left side only touch in part, so that the molars are about all that remain for mastication.

One of the superior centrals and the canine standing beside it, owing to the early loss or non-appearance of the lateral, are not affected in the least on the labial surface; but the ends of the teeth are wasted away in the same manner as the other front teeth.

I have been able to cover the surfaces affected from time to time, some of them with gold, and for others I have used oxyphosphate with a small quantity of alloy filings mixed with it, as I find that fillings of this material wear longer when the filings are added to it. The disease has continued, however, to affect the tooth-structure around the edges of the fillings, showing its persistence.

The patient is between seventy-five and eighty years of age, and is of at least more or less a gouty and rheumatic diathesis. When he first came into my hands, some twelve years ago, I attributed much of the loss of the enamel on the labial surfaces to the excessive use of the brush, as he gave me to understand that he was a hard brusher; but it has been clearly shown since that the brush was not the cause of the trouble.

I may say that I have never known a case in which the disease in question affected the lingual surfaces of the teeth.

*Dr. Remington.*—I saw a case three days ago where it attacked the lingual surfaces.

*Dr. Bulkley.*—I think the point is that we get the uric-acid indication long before we get the more active manifestations of gout. The contracted kidney is one of the latest links in the chain although apoplexy, etc., comes after that, of course. We get certain cases of neuralgia which are gouty. I believe the erosion of the teeth can come long before the person has any pain in the toes or in the joints. If they will modify the diet before the severely acid state is produced, then the action ceases.

*Dr. Howe.*—Dr. Jarvie sent word that he was very anxious to be here to-night, but could not come. He sent a series of three models of the effects of erosion on the teeth in a certain mouth. They show the progress of erosion in one mouth from 1882 to 1889. He says the lady is now dead, but that he watched the case all the way through, and that he particularly inquired, as I understand it, for rheumatism or gout, or anything pointing in that direction, and that there never was any history of any such thing in her case. These models are here, and any one interested in them can look at the models.

*Dr. Littig.*—Although we may not see that there has been any particular indication of gout, I never saw a case of erosion that there was not that peculiar, nervous condition of the patient that is the forerunner of an organic disease. I think that is one of the points that has been overlooked to-night.

Adjournment.

JOHN I. HART, D.D.S.,
*Editor New York Odontological Society.*

## Figures and Tables

**Figure f1:**